# Microbial anabolic and catabolic utilization of hydrocarbons in deep subseafloor sediments of Guaymas Basin

**DOI:** 10.1093/femsec/fiae093

**Published:** 2024-07-02

**Authors:** Toshiki Nagakura, Yuki Morono, Motoo Ito, Kai Mangelsdorf, Stefanie Pötz, Ellen Schnabel, Jens Kallmeyer

**Affiliations:** GFZ German Research Centre for Geosciences, Section Geomicrobiology, Telegrafenberg, 14473 Potsdam, Germany; JAMSTEC Japan Agency for Marine-Earth Science and Technology, Kochi Institute for Core Sample Research, 200 Monobe Otsu, 783-8502 Nankoku, Kochi, Japan; JAMSTEC Japan Agency for Marine-Earth Science and Technology, Kochi Institute for Core Sample Research, 200 Monobe Otsu, 783-8502 Nankoku, Kochi, Japan; GFZ German Research Centre for Geosciences, Section Organic Geochemistry, Telegrafenberg, 14473 Potsdam, Germany; GFZ German Research Centre for Geosciences, Section Organic Geochemistry, Telegrafenberg, 14473 Potsdam, Germany; GFZ German Research Centre for Geosciences, Section Geomicrobiology, Telegrafenberg, 14473 Potsdam, Germany; GFZ German Research Centre for Geosciences, Section Geomicrobiology, Telegrafenberg, 14473 Potsdam, Germany

**Keywords:** anabolism, catabolism, Guaymas Basin, hydrocarbon, NanoSIMS

## Abstract

Guaymas Basin, located in the Gulf of California, is a hydrothermally active marginal basin. Due to steep geothermal gradients and localized heating by sill intrusions, microbial substrates like short-chain fatty acids and hydrocarbons are abiotically produced from sedimentary organic matter at comparatively shallow depths. We analyzed the effect of hydrocarbons on uptake of hydrocarbons by microorganisms via nano-scale secondary ion mass spectrometry (NanoSIMS) and microbial sulfate reduction rates (SRR), using samples from two drill sites sampled by IODP Expedition 385 (U1545C and U1546D). These sites are in close proximity of each other (ca. 1 km) and have very similar sedimentology. Site U1546D experienced the intrusion of a sill that has since then thermally equilibrated with the surrounding sediment. Both sites currently have an identical geothermal gradient, despite their different thermal history. The localized heating by the sill led to thermal cracking of sedimentary organic matter and formation of potentially bioavailable organic substrates. There were low levels of hydrocarbon and nitrogen uptake in some samples from both sites, mostly in surficial samples. Hydrocarbon and methane additions stimulated SRR in near-seafloor samples from Site U1545C, while samples from Site U1546D reacted positively only on methane. Our data indicate the potential of microorganisms to metabolize hydrocarbons even in the deep subsurface of Guaymas Basin.

## Teaser

Hydrothermally influenced sediments from Guaymas Basin harbor microorganisms that metabolize hydrocarbons via both anabolic and catabolic pathway.

## Introduction

The deep subseafloor biosphere harbors vast amounts of prokaryotes, their number is thought to be approximately the same as in soil and seawater (Kallmeyer et al. 2012). In addition, microorganisms in deep subsurface sediments are metabolically active (D’Hondt et al. 2004, Schippers et al. 2005) or at least revivable (Morono et al. 2011, 2020, Trembath-Reichert et al. 2017). Because of its great amounts of biomass, the deep biosphere is considered to play a vital role in the global cycling of elements (Parkes et al. 2014).

Guaymas Basin, located in the Gulf of California (Mexico), is characterized by strong hydrothermal activity due to seafloor spreading (Kawka and Simoneit 1987). Due to high productivity in its surface waters and in parts high terrigenous sediment input, organic-rich sediment accumulates at rates exceeding 1 mm year^−1^ (Calvert 1966, Curray et al. 1979, Teske et al. 2021a). In areas with steep geothermal gradients like Guaymas Basin or Nankai Trough off Japan, bioavailable organic substrates like volatile fatty acids are produced by geothermal degradation (pyrolysis) of macromolecular sedimentary organic matter (kerogen) already at shallow depths (Kawka and Simoneit 1994, Horsfield et al. 2006, Teske et al. 2014). In addition, laboratory experiments showed that acetate is produced when heating sediment to temperatures in the mesophilic to thermophilic range (Wellsbury et al. 1997). These findings support the notion that *in situ* production of organic substrates can support life in the deep subsurface biosphere. The supply of carbon sources in Guaymas Basin is therefore expected to be relatively high and diverse (review in Edgcomb et al. 2022).

Recent technological developments, e.g. deep drilling with contamination control for recovery of samples suitable for microbiological and molecular biological analyses, as well as sensitive techniques e.g. for detections of ultra-low abundances of microbial cells, made it possible to study microbial communities and metabolic activities in the deep biosphere (Colwell and D’Hondt 2013, Morono and Inagaki 2016, Kallmeyer 2017, Morono 2023). Additionally, to elucidate the metabolic activity of microorganisms in various environments, many molecular biological or chemical analytical techniques are used. Nano-scale secondary ion mass spectrometry (NanoSIMS) is a powerful tool to determine solid surface compositions (e.g. minerals and cells) on the single-cell level because it is capable of nm-scale resolution (e.g. Ito and Messenger 2008, Kubota et al. 2014, Morono et al. 2020). This technique, therefore, allows quantification of uptake of stable-isotope labeled substrates at the single-cell level (Lechene et al. 2006, Wagner 2009). Recent studies used NanoSIMS to detect viable cells in 20 million-year-old coal bed sediment from depths of over 2000 meters below seafloor (mbsf) off the Shimokita Peninsula, Japan, and in 100 million-year-old sediment of the oligotrophic South Pacific Gyre (Trembath-Reichert et al. 2017, Morono et al. 2020). Both environments are characterized by extremely low cell abundances (10^4^–10^0^ cells cm^−3^ below 1500 mbsf and 10^6^–10^2^ cells cm^−3^ down to 100 mbsf, respectively) (Inagaki et al. 2015, Trembath-Reichert et al. 2017, Morono et al. 2020).

In anoxic subsurface sediment, after other thermodynamically more efficient electron acceptors (O_2_, NO_3_^−^, Mn(IV), and Fe(III)) are depleted, sulfate reduction becomes the quantitatively dominant organic matter mineralization process (Jørgensen 1982, 2000, Parkes et al. 2014). The biodegradation of hydrocarbons under anaerobic conditions and the respective metabolic strategies have already been studied extensively (e.g. Meckenstock and Mouttaki (2011) and references therein). Sulfate reducers can also metabolize a wide variety of carbon sources including aliphatic or aromatic hydrocarbons (Reuter et al. 1994, Coates et al. 1996, Shin et al. 2019). Sulfate reduction fueled by organic matter is commonly termed organoclastic sulfate reduction. Below the zone of organoclastic sulfate reduction, sulfate reduction can be coupled with methane oxidation (methanotrophic sulfate reduction, or anaerobic oxidation of methane; AOM) through a consortium of archaeal methanotrophs and sulfate-reducing bacteria (Iversen and Jorgensen 1985, Hoehler et al. 1994, Boetius et al. 2000). This process is usually restricted to the relatively narrow depth interval where downward diffusing sulfate and upward diffusing methane overlap, the so-called sulfate–methane transition zone (SMTZ). Sulfate is not fully depleted in the SMTZ and remains at low µM levels due to the reoxidation of sulfide via a cryptic iron-driven sulfur cycle (Holmkvist et al. 2011).

Pyrolysis of sedimentary organic matter leads to the formation and release of a wide range of hydrocarbons in Guaymas Basin that fuel microbial activity in these sediments (Teske et al. 2014). While the microbiology of Guaymas Basin’s surface sediments have been studied for decades, the potential of anabolic and catabolic metabolisms by microorganisms living in the deep subsurface of this ecosystem has not been studied due to a lack of suitable samples. Our study aims to elucidate anaerobic microbial metabolic activities involving hydrocarbons in deep subseafloor sediment of Guaymas Basin by addressing the following questions: (1) which types of hydrocarbons are assimilated by microorganisms and (2) do hydrocarbons influence microbial catabolic activity, i.e. is microbial sulfate reduction coupled to hydrocarbon degradation? The first question is addressed through the detection of uptake of stable isotope-labeled aliphatic/aromatic hydrocarbons as well as methane, using NanoSIMS. To address the second question, we quantified the effect of aliphatic/aromatic hydrocarbon or methane addition on microbial sulfate reduction via incubation experiments using ^35^SO_4_^2−^ radiotracer. Most hydrocarbons used for these experiments (*n*-decane, *n*-hexadecane, *n*-icosane, naphthalene, anthracene, phenanthrene, and methane) were detected in Guaymas Basin sediments, the other two hydrocarbons (squalene and benzene) were not measured but expected to exist (Edgcomb, personal communication).

## Materials and methods

### Sampling

The samples were recovered in 2019 during the IODP Expedition 385; Guaymas Basin Tectonics and Biosphere (Teske et al. 2021a, Nagakura et al. 2022). Immediately after sampling, the drilled whole round core samples were placed in nitrogen-filled gas-tight bags and stored at 4°C until further subsampling in the home lab. For our study, we used cores from U1545C and U1546D (Table 1). These two sites are about 1.1 km apart from each other. Stratigraphy and sediment composition are almost identical at both sites, but sediments at Site U1546D were affected by a sill intrusion. (From this section, Site U1545C is called “the nonsill site” and Site U1546D is called “the sill site”.) However, since the temperature gradients at both sites are almost identical (225°C km^−1^ at the nonsill site and 221°C km^−1^ at the sill site), we can assume that heat from the sill has already dissipated (Teske et al. 2021a, Nagakura et al. 2022). Table 1 shows the depth and temperature data of the core samples used for this study. The samples were selected from a wide range of temperatures (4°C–63°C) at similar depths of these two sites. Additionally, we chose one sample from each site that is located near the SMTZ. The measured data in this paper is comparable to the previous data (Nagakura et al. 2022). As the measurements in Nagakura et al. (2022) were performed as soon as possible after the expedition, the change in microbial community during the sample conservation at 4°C would be minimal.

**Table 1.** Depth and temperature data of the samples from the IODP Expedition 385 nonsill site (U1545C) and sill site (U1546D). The SMTZ at the nonsill site and the sill site are around 40 mbsf and 110 mbsf, respectively. The incubation temperatures were within ±2°C of their *in situ* temperatures.

Site U1545C (nonsill site) 27°38.2420′ N 111°53.3290′ W					Site U1546D (sill site) 27°37.8943′ N 111°52.7812′ W				
Core number	Depth (mbsf)	*In situ* temperature (°C)	Incubation temperature for radioisotope experiment (°C)	Incubation temperature for stable isotope experiment (°C)	Core number	Depth (mbsf)	*In situ* temperature (°C)	Incubation temperature for radioisotope experiment (°C)	Incubation temperature for stable isotope experiment (°C)
1	2.0	4.2	4	4	1	2.1	4.3	4	4
6	44.4	13.8	14	15	6	43.8	13.5	14	13
7	54.6	16.1	17	−	14	104.1	26.8	28	−
12	103.9	27.2	28	−	15	114.4	29.1	31	−
14	123.0	31.5	31	32	16	123.8	31.2	31	31
27	185.3	45.5	45	−	23	190.7	46.0	45	−
43	260.7	62.5	63	62	37	261.5	61.7	62	62

### Organic geochemical analyses of hydrocarbons in subsurface sediments

In order to obtain additional information on the geochemical habitat in which the microbial cells are living, and to see whether the natural hydrocarbon compositions differ between sites, we analyzed the aliphatic organic compounds using gas chromatography–mass spectrometry (GC–MS) and nitrogen, sulfur, and oxygen (NSO) containing compounds using Fourier transform–ion cyclotron resonance–mass spectrometry (FT–ICR–MS).

Freeze-dried sediment sample aliquots of 0.3–0.7 g were extracted with 200 ml dichloromethane/1% methanol using Soxhlet extraction (Luque de Castro and Priego-Capote 2010). Afterwards, the extracts were concentrated using a TurboVap500 system. Subsequently, the asphaltenes were removed by dissolving the extracts in 250 µl DCM/MeOH (99:1 v/v) and adding a 40-fold excess of *n*-hexane, leading to the precipitation of *n*-hexane-insoluble substances. For compound quantification, four internal standards were added: 5α-androstane, ethylpyrene, 5α-androstan-17-one, and erucic acid. Using *n*-hexane, we separated the maltene fraction (*n*-hexane soluble compounds) by medium-pressure liquid chromatography (Radke et al. 1980) into an aliphatic, aromatic, and NSO fraction.

The aliphatic fraction was measured on a Trace Gas Chromatograph 1310 (Thermo Scientific) coupled to a TSQ 9000 mass spectrometer (Thermo Scientific). The gas chromatograph was equipped with a cold injection system operating in splitless mode and a SGE BPX 5 fused-silica capillary column (50 m length, 0.22 mm ID, and 0.25 µm film thickness) using the following temperature conditions: initial temperature 50°C (1 min isothermal), heating rate 3°C min^−1^ to 310°C, held isothermally for 30 min. Helium was used as carrier gas with a constant flow of 1 ml min^−1^. The injector temperature was programmed from 50°C to 300°C at a rate of 10°C s^−1^. The MS operated in the electron impact mode at 70 eV. Full-scan mass spectra were recorded from m/z 50 to 650 at a scan rate of 1.5 scans s^−1^.

The NSO fraction was measured in methanol and toluene (1:1, v/v) at a concentration of 25 µg ml^−1^ negative ion electrospray ionization (ESI) mode using a 12 Tesla FT–ICR–MS solariX upgraded with a ParaCell equipped with an Apollo II ESI source (both from Bruker Daltonik GmbH, Bremen). Nitrogen was used as drying gas at a flow rate of 4.0 l min^−1^ and a temperature of 220°C and as nebulizing gas with 1.4 bar. The sample solutions were infused at a flow rate of 150 µl h^−1^. The capillary voltage was set to 3000 V and an additional collision-induced dissociation voltage of 60 V in the source was applied to avoid cluster and adduct formation. Ions were accumulated in the collision cell for 0.05 s and transferred to the ICR cell within 1 ms. Spectra were recorded in broadband mode using 8 megaword data sets. For each mass spectrum, 200 scans were accumulated in a mass range from m/z 147 to 1000. Sine-bell apodization was applied prior to the Fourier transformation to produce the frequency domain data, which was then converted to the mass spectrum.

Internal quadratic recalibration was performed with a standard deviation error < 0.009. Signals with signal-to-noise ratio ≥6 were included in data assessment. Formula assignment was done using the elemental ranges C_5–100_H_5–200_N_0–2_O_0–10_S_0–2_Na_0–1_ using a combination of Bruker Analysis, Microsoft Excel, and R. Venn analysis was done in R.

### Sample preparation for stable-/radioisotope experiments

The following procedures were applied to both the radioisotope and stable-isotope experiments described in the following sections. All materials in contact with the sample were either autoclaved or combusted (400°C for 4 h). All sample handling was carried out inside a nitrogen-filled anoxic glovebox. In the anoxic glovebox, 10 g of sediment was placed into the precombusted glass crimp vial (volume: 30 ml) at ca. 6°C and mixed with anoxic artificial seawater medium to form a slurry. Note that the outer sediment in whole round cores was not used to avoid contamination. The vial was closed with a thick black butyl rubber stopper without any headspace. For the incubation at approximate *in situ* pressure (ca. 25 MPa) and temperature (Table 1), we placed the vials in stainless steel high-pressure cylinders. Because of the low compressibility of water, the flexibility of the rubber stopper was enough to transfer the pressure of the high-pressure cylinder into the vial. Since we realized that the thick rubber stoppers did not work properly to transfer the pressure at low temperatures, we cut off the bottom 5 mm of the stoppers to make them thinner, and hence more flexible.

### Quantification of hydrocarbon and inorganic nitrogen uptake via NanoSIMS

We aimed to observe hydrocarbon and inorganic nitrogen uptake by incubating sediment samples with stable-isotope-labeled hydrocarbons and ammonium chloride and analyze them using NanoSIMS.

#### Medium preparation for stable-isotope analyses

The composition of the medium was the same as in Nagakura et al. (2022), but slightly modified for the stable isotope uptake analysis with; 0.2 g KH_2_PO_4_, 0.225 g ^14^NH_4_Cl, 0.025 g ^15^NH_4_Cl (for nitrogen uptake analysis), 25 g NaCl, 0.5 g MgCl_2_ × 6H_2_O, 0.5 g KCl, 0.15 g CaCl_2_ × 2H_2_O, and 0.71 g Na_2_SO_4_ mixed with 1 l of MilliQ water. 3 ml of 0.1% resazurin was added to the medium and autoclaved. 5 ml of Na_2_S solution (0.12 g Na_2_S in 10 ml MilliQ water) and 5 ml of NaHCO_3_ solution (0.84 g NaHCO_3_ in 10 ml MilliQ water) were added to the medium and flushed with N_2_/CO_2_ gas for ca. 2 h. The medium was then stored in autoclaved serum bottles flushed with N_2_/CO_2_ gas until use.

#### Sample incubation with stable isotope substrates

Similar to the SRR measurements (see below), we separated the samples for quantification of anabolic activity (i.e. uptake) via NanoSIMS into two groups, (a) uptake of benzene (C_6_H_6_) and *n*-hexadecane (C_16_H_34_) and (b) uptake of methane. Both types of samples were amended with ^15^N-ammonium chloride to monitor uptake of inorganic nitrogen. (a) Each 10% (w/v of acetone) hydrocarbon stock solution (*n*-hexadecane and benzene) was prepared after Widdel and Bak (1992). The *n*-hexadecane stock solution consisted of 90wt% of *n*-hexadecane and 10wt% of fully deuterated *n*-hexadecane-d_34_. The benzene stock solution consisted of 80wt% of benzene and 20wt% of benzene-^13^C_6_. When the medium was added to the glass vial containing the sample, 400 µl of each hydrocarbon stock solution was added as well, and the sample vial was closed with a black butyl rubber stopper and crimped. The stock solutions were added to the sample vials at room temperature to keep the hydrocarbons dissolved in acetone. In addition, a 3-ml syringe including 0.5 ml of the medium was placed into each vial to avoid breakage of the glass vial upon pressurization. The samples were preincubated overnight in the anoxic glovebox to allow the microorganisms to adjust to the new conditions. (b) For the incubation with methane, the sediment samples were mixed with the medium and preincubated in the anoxic glovebox overnight. After preincubation, 10 ml of gaseous methane, consisting of 80v% ^12^C-methane and 20v% of ^13^C-methane was injected into the sample vial. For both types of incubations we also prepared two killed controls (KCs), which were mixed with 20% ZnAc instead of media. Other conditions were the same as the samples mentioned above; one KC was prepared with hydrocarbons (a), and the other one with methane (b). Since there is no microbial activity in KCs, the labeled substrates are not incorporated and can be used for the statistical criterion (see below).

The samples were put into high-pressure cylinders and they were incubated in a HPTGB system (Kallmeyer et al. 2003, Nagakura et al. 2022) at *in situ* temperature (Table 1) and pressure (ca. 25 MPa) for 42 days.

#### Sample fixation

At the end of the incubation, samples were fixed in phosphate-buffered saline (PBS) solution and paraformaldehyde (PFA) solution. The solutions were prepared as 10x PBS and 9.33% PFA. For 10x PBS solution, 79.4 g NaCl, 1,9 g KCl, 11.4 g Na_2_HPO_4_, and 2.6 g KH_2_PO_4_ were mixed in 1 l MilliQ (pH 7.2). The solution was then autoclaved and stored at room temperature. For 9.33% PFA solution, 28 g of PFA was added to 260 ml MilliQ water and heated to ca. 60°C. As PFA only dissolves in alkaline conditions, 1 N NaOH was added until the PFA was dissolved. The solution was then cooled down and 30 ml of 10x PBS was added. HCl was added to adjust the pH to 7.2. To maintain consistent osmotic conditions, we also added 5.1 g NaCl. The solution was brought to its final volume of 300 ml by adding MilliQ water, then the solution was sterile filtered (0.2 µm pore size). When the PFA solution is mixed with the incubation media, it has a final concentration of ca. 4%.

Upon removal of the samples from the HPTGB, the sample vials were opened and the sediment and medium were immediately transferred into 50 ml centrifuge tubes including 15 ml of 9.33% PFA solution and stored for 22 h at 4°C. After this fixation step, the samples were centrifuged at 2500 ×*g* for 15 min and the supernatant was discarded. Afterwards, the samples were washed twice with 1x PBS. Once the samples were fixed, they were preserved in PBS/ethanol (1:1, v/v) and kept at −20°C until analysis at JAMSTEC in Kochi, Japan.

#### Cell detaching and sorting

At JAMSTEC, the following procedures (modified from Morono et al. 2013) were performed for detaching cells from sediments.

1. 0.5 ml of the sample slurries were transferred into new 15 ml centrifuge tubes and mixed with 1 ml of 2.5% NaCl.
2. Samples were centrifuged at 5000 × *g* for 10 min and the supernatants were discarded.
3. The sediment pellets were mixed with 2.7 ml of prefiltered 3x PBS, 0.5 ml NaCl, 0.4 ml detergent mix (D-mix) (Kallmeyer et al. 2008), and 0.4 ml methanol.
4. The suspensions were shaken at 500 r/m for 1 h with a shaker (ShakeMaster, BMS Biomedical Science), followed by sonication (Bioruptor, Cosmo Bio Co., Ltd.) for 20 min (20 cycles of sonication at 200 W for 30 s with 30 s intervals).
5. The samples were carefully put onto the top surface of the following density gradient containing the following solutions: from surface to bottom in 15 ml centrifuge tubes; 4 ml of 30% (v/v) Nycodenz, 4 ml of 50% (v/v) Nycodenz, 4 ml of 80% (v/v) Nycodenz, and 4 ml of 67% (v/v) sodium polytungstate.
6. The tube was centrifuged for 1 h at 10 000 × *g* and 4°C.
7. The supernatants were collected and transferred into new 15 ml centrifuge tubes.
8. 5 ml of NaCl was added to the sediment and resuspended.
9. The slurry was centrifuged at 6000 × *g* for 15 min and the supernatant was discarded.
10. The rest was mixed with 2.2 ml of NaCl, 0.4 ml of D-mix, and 0.4 ml of methanol.
11. The mixed solution was shaken at 500 r/m for 10 min.
12. The sample was then sonicated for 20 min.
13. The sample was placed gently onto the gradient layer solution in 15 ml centrifuge tubes.
14. The tube was centrifuged at 10 000 × *g* for 1 h.
15. The supernatant was carefully recovered and stored with the supernatant collected before.

The cells in the supernatant were collected by filtration through an Anodisc^TM^ 25 aluminum oxide filter (pore size 0.2 µm). 500 µl of 1x TE buffer was also placed on Anodisc to wash out the density-gradient compounds. After removing the solution and stopping the vacuum pump, 110 µl of 40x diluted SYBR Green I (Thermo Fischer Scientific) was immediately put onto the filter and left for 10 min. After 10 min, 500 µl of 1x TE buffer was poured onto the Anodisc membrane while vacuuming to wash the membrane. Right after the final drop of the liquid had passed through the membrane, the membrane was immediately put in a 50-ml centrifuge tube containing 5 ml of 1x TE buffer, placing the side containing the cells facing down. The tube containing the filter was then sonicated twice for 30 s each at 200 W. Then the suspension was stored at 4°C.

The stained cells were sorted by a cell sorter (MoFlo XDP, Beckman Coulter). Cells were directly sorted onto an indium tin oxide (ITO)-coated membrane. Approximately, 10 000 cells were sorted on the ITO membrane. For the sample from the nonsill site Core 16 incubated with benzene and *n*-hexadecane, 30 000 cells were sorted. The area where the cells were sorted was marked by laser microdissection (LMD6000; Leica Microsystems).

#### Analysis of hydrocarbon uptake with NanoSIMS

The sorted microbial cells on the ITO-coated membrane (Morono et al. 2020) were analyzed with the JAMSTEC NanoSIMS 50L ion microprobe (AMETEK Co. Ltd, CAMECA BU). In the samples, incubated with benzene and *n*-hexadecane, ^1^H^−^ and ^2^H^−^ were analyzed after the analysis of carbon and nitrogen isotopes. The analytical procedures were described elsewhere (e.g. Morono et al. 2020). In brief, a focused primary positive Cs ion beam of ∼1.5 pA was used for carbon and nitrogen isotopic analyses, and approximately ∼6 pA was used for hydrogen isotopic analysis, rastered over 24 µm × 24 µm areas on the samples. Each analysis was initiated after stabilization of the secondary ion beam intensity following several minutes of presputtering with a relatively strong primary ion beam current (∼20 pA). For carbon and nitrogen isotopic analysis, images of ^12^C^−, 13^C^−, 16^O^−, 12^C^14^N^−, 12^C^15^N^−^, and ^32^S^−^ were acquired simultaneously in multidetection with six electron multipliers (EMs) at a mass resolving power of ∼9000, sufficient to separate all relevant isobaric interferences (^12^C^1^H on ^13^C and ^13^C^14^N on ^12^C^15^N). For hydrogen isotopic analysis, images of ^1^H^−, 2^H^−^, and ^12^C^−^ were acquired using three EMs in multidetection mode at a mass resolving power of ∼3000. Each analysis consisted of the same area, which individual images consisting of 256 pixels × 256 pixels. The dwell times were 2 ms pixel^−1^ (131.072 s scan^−1^) for the carbon and nitrogen isotopic analyses and 5 ms pixel^−1^ (327.68 s scan^−1^) for the hydrogen isotopic analysis.

#### Examination of isotope abundance ratio with OpenMIMS

After the NanoSIMS analysis was performed, the data was saved in IM files. To open and check the NanoSIMS images, the OpenMIMS plugin in the application ImageJ of Fiji software was used. Each of the vertically stacked spattered planes was aligned to correct the drift during the acquisition of each plane image and integrated into an image.

**Figure 1.**
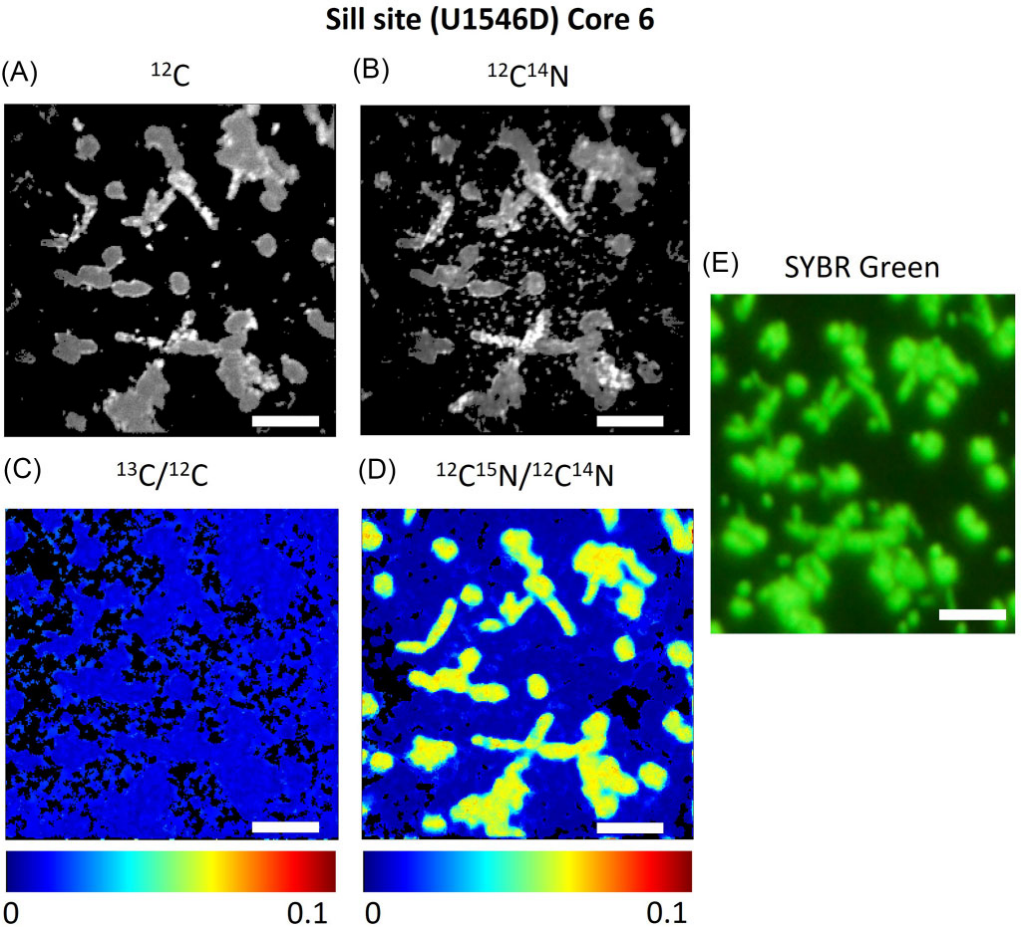
NanoSIMS isotope images and fluorescence microscopy images of the sample Core 6 at the sill site (U1546D), incubated with methane. (A) ^12^C isotope images analyzed with NanoSIMS. (B) ^12^C^14^N isotope images analyzed with NanoSIMS. (C) Isotope ratio image of ^13^C/^12^C, (D) isotope ratio image of ^12^C^15^N/^12^C^14^N, and (E) fluorescence microscopy images. The cells were dyed with SYBR Green I. The color code with numbers (C and D) indicates the isotope ratios. Scale bars in each panel represent 5 µm. The isotope images were processed with ImageJ.

To identify the cellular regions, the NanoSIMS images were compared to the fluorescence microscopy images, which were taken before (Fig. 1). We were able to match the isotope images and the fluorescence microscopy image in about half of the samples.

Cells shown in the isotope images were marked as regions of interest (ROI) and the isotope abundance of each ROI was thereby calculated. ^13^C/(^12^C+^13^C), ^12^C^15^N/(^12^C^14^N+^12^C^15^N), or ^2^H/(^1^H+^2^H) isotope abundance ratios were calculated to examine if the cells assimilated the isotopes. ROI were drawn based on the following criteria: (1) ROI were drawn based on the clear isotope signals in ^12^C images. When the signals are clearly detected, ROI were drawn also based on the ^13^C images or ^12^C/^15^ N images (heavy isotope images). (2) If at least one of the isotope abundance ratios was zero, the ROI was excluded. These criteria were also applied to the samples matching the NanoSIMS and the fluorescence microscopy images.

Since the calculated values are not absolute values but relative values, the ROI in each analysis were standardized by the “blank” ROI drawn at a membrane region without any cells. This blank region is regarded as the natural abundance ratio of each isotope (^13^C/(^12^C+^13^C) = 1.06%, ^15^ N/(^14^N+^15^ N) = 0.4%, and ^2^H/(^1^H+^2^H) = 0.0115%; Trivedi et al. 2016). The isotope abundance ratios were calculated as follows (here presented for carbon as an example).


\begin{eqnarray*}
{\textrm{Isotope abundance ratio}}\,\,\left( {{\mathrm{C}},\% } \right) = {}^{13}{\mathrm{C}}/\left( {{}^{12}{\mathrm{C}} + {}^{13}{\mathrm{C}}} \right) \times 100.
\end{eqnarray*}

This calculation was also done for the KCs to obtain the average and standard deviation values for the statistical threshold.


\begin{eqnarray*}
\begin{array}{@{}l@{}} {\textrm{Statistical threshold}}\left( - \right) \\ = \left( {{\textrm{Average of the isotope abundance ratio of KC}}} \right) \\ + 3 \times \left( {{\textrm{Standard deviation of the isotope abundance ratio of KC}}} \right)\!. \end{array}
\end{eqnarray*}

This calculation assumes that the ROI values of KCs are normally distributed. The values were then converted to the isotope abundance ratios and described in percentages.

Since ^13^C/(^12^C+^13^C) and ^12^C^15^N/(^12^C^14^N+^12^C^15^N) were measured separately from ^2^H/(^1^H+^2^H), we tried to match the ROI to those based on the carbon, nitrogen, and hydrogen isotope images as much as possible. Based on the position of cells in each isotope image, the cells were manually matched to each other and summarized in the 3D plot.

### Radioisotope experiment for the measurements of SRR

In order to quantify the effect of hydrocarbon addition on SRR, we incubated the samples with ^35^SO_4_^2−^ radiotracer and a mixture of various hydrocarbons or methane. The SRR measurements without hydrocarbon additions were already presented by Nagakura et al. (2022).

#### Medium preparation

Medium composition and preparation for SRR measurements are the same as in Nagakura et al. (2022): 0.2 g KH_2_PO_4_, 0.25 g NH_4_Cl, 25 g NaCl, 0.5 g MgCl_2_ × 6H_2_O, 0.5 g KCl, 0.15 g CaCl_2_ × 2H_2_O, and 0.71 g Na_2_SO_4_ were mixed with 1 l of MilliQ water. 3 ml of 0.1% resazurin was added to the medium and autoclaved. After autoclaving, 5 ml of Na_2_S solution (0.12 g Na_2_S in 10 ml MilliQ water) and 5 ml of NaHCO_3_ solution (0.84 g NaHCO_3_ in 10 ml MilliQ) were added to the medium and flushed with N_2_/CO_2_ gas for ca. 2 h. The medium was then stored in precombusted crimp bottles flushed with N_2_/CO_2_ gas until use.

#### Sample preparation and incubation with 35S sulfate and hydrocarbon substrates

We quantified SRR with two different hydrocarbon additions, (a) a mixture of eight hydrocarbons and (b) methane. (a) We prepared eight stock solutions, each containing one hydrocarbon in a concentration of 10% (w/v in acetone) (Widdel and Bak 1992). The eight stock solutions contained the aliphatic hydrocarbons *n*-decane (C_10_H_22_), *n*-hexadecane (C_16_H_34_), *n*-icosane (C_20_H_42_), squalene (C_30_H_50_), as well as the aromatic compounds benzene (C_6_H_6_), naphthalene (C_10_H_8_), anthracene (C_14_H_10_), and phenanthrene (C_14_H_10_). In the anoxic glovebox, the medium and 100 µl of each hydrocarbon stock solution were added to the sample vial containing the sediment. The hydrocarbon addition into the sample vial was performed at room temperature to keep hydrocarbons dissolved in acetone. The sample vial was then closed with a black butyl rubber stopper and crimped. Since *n*-icosane, anthracene, and phenanthrene did not dissolve completely in acetone, they were added as suspensions. The samples were kept in the anoxic glovebox overnight at ca. 6°C. (b) For the incubation with methane, the medium was added to the glass vial containing the sample. The sample vial was closed with a black butyl rubber stopper, crimped, and kept in the anoxic glovebox overnight at ca. 6°C. The next day, a syringe containing 10 ml of methane was connected to the sample vial with an injection needle through the rubber stopper. All samples were prepared in duplicates, as triplicates were not possible due to limited amounts of sample material. Table 1 shows the incubation temperature of each sample. Additionally, each run of incubations included KCs and medium controls (MCs). For KCs, sediment was mixed with 20% ZnAc instead of medium and either 100 µl of each hydrocarbon stock solution or 10 ml of methane. MCs contained only media. These controls were used to confirm that sulfate reduction was carried out biologically and that neither the medium (MCs) nor the sediment (KCs) causes abiotic sulfate reduction, and thus interferes with the quantification of biological turnover. After the preincubation, the samples, as well as the KCs and MCs, were injected with 5 MBq ^35^SO_4_^2−^ radiotracer and incubated in our high-pressure thermal gradient block (HPTGB) (Kallmeyer et al. 2003, Nagakura et al. 2022) at *in situ* temperatures and pressure (ca. 25 MPa) for 10 days. In short, the HPTGB consists of a thermally insulated 1.5 m-long aluminum block, the ends of the block can be heated or cooled individually to achieve a thermal gradient. There are three lines of 15 holes each in the block, each hole can house one high-pressure cylinder. Every line has its own pressure system, so we can incubate samples at up to 45 different pressure/temperature conditions. After incubation, the high-pressure cylinders were depressurized, and the sample vials were removed from the cylinders. Upon opening the glass vials, the samples were immediately poured into 50 ml centrifuge tubes containing 5 ml of 20% ZnAc. To ensure quantitative transfer of sample and medium, we rinsed the glass vials twice with 5 ml of 20% ZcAc each, which was added to the same centrifuge tube. Samples were stored at −20°C until analysis.

#### Sample distillation and scintillation counting followed by SRR calculation

All inorganic reduced sulfur species (total reduced inorganic sulfur, TRIS), which also contain the microbially produced radiolabeled sulfide, were separated from the sample by cold chromium distillation (Kallmeyer et al. 2004). After thawing the samples, they were centrifuged for 10 min at 2500 × *g*. To quantify the total radioactivity, 50 µl of the supernatant was transferred to a scintillation vial and mixed with 4 ml of scintillation cocktail (Rotiszint® eco plus LSC-Universalcocktail, Carl Roth). The remainder of the supernatant was carefully decanted off and the sediment sample was mixed with 15 ml of *N, N*-dimethylformamide and quantitatively transferred to a glass distillation flask. A magnetic stir bar was put into the flask and set at 400 r m^−1^ to ensure complete mixing of the sample and chemicals. The flask was flushed with N_2_ to maintain anoxic conditions. After 10 min of N_2_ flushing, 8 ml of 6 M HCl and 15 ml of 1 M chromium (II) chloride solution were added through a reaction port to convert all reduced sulfur species in the sediment sample to gaseous H_2_S. The H_2_S was driven out of the solution by the constant stream of N_2_ gas and led through a first trap filled with 7 ml of citric acid solution (19.3 g of citric acid and 4 g of NaOH in 1 l MilliQ water; pH 4) to trap all aerosols, potentially containing unreacted ^35^S-sulfate, before reaching a second trap filled with 7 ml of 20% ZnAc solution in which the H_2_S is quantitatively converted to solid ZnS. To avoid overflowing of the zinc acetate trap, a few drops of silicon-based antifoam were added. The distillation lasted for 2 h. Normally, only 5% ZnAc solution is used for the traps, but the amounts of sulfide in the sample require higher concentrations of ZnAc to ensure the trapping of all sulfide. To avoid the possible interference by high concentrations of acetate with the scintillation cocktail, the 20% ZnAc solution was centrifuged at 2500 × *g* for 10 min and the supernatant was discarded. The ZnS pellet was resuspended with 5% ZnAc and the total volume was adjusted to 7 ml. The ZnS suspension was then quantitatively transferred into a 20-ml plastic scintillation vial and mixed with 8 ml of scintillation cocktail. Distillations were carried out in batches of 10 samples plus one distillation blank (DB), containing only a few drops of nonradioactive ZnS carrier. Counter blanks contained only 7 ml of 5% ZnAc solution and 8 ml of the scintillation cocktail. MCs and DBs were then directly transferred into plastic scintillation vials and mixed with 8 ml of scintillation cocktail.

Radioactivity was quantified using a HIDEX 600 SL liquid scintillation counter (HIDEX Oy) with guard scintillator. Before the vials were placed into the counter, they were vortexed to ensure complete mixing of the sample and scintillation cocktail, and the surface of the vial was wiped with a cleaning wipe (Kimtech Science) moistened with 70% ethanol to remove any potential contamination on the surface of the vial.

SRR was calculated as follows:


\begin{eqnarray*}
{\mathrm{SRR}} = {\left( {{\mathrm{S}}{{\mathrm{O}}_4}^{2 - }} \right)_{{\mathrm{TOT}}}}/{{\mathrm{V}}_{{\mathrm{SED}}}} \times {{\mathrm{a}}_{{\mathrm{TRIS}}}}/{{\mathrm{a}}_{{\mathrm{TOT}}}} \times 1/{\mathrm{t}} \times 1.06,
\end{eqnarray*}

where SRR is calculated in pmol cm^−3^ d^−1^, (SO_4_^2−^)_TOT_ is the total amount of sulfate in the sample (sulfate in the sediment + sulfate in the medium; pmol), V_SED_ is the volume of the sediment sample (cm^3^), a_TRIS_ is the radioactivity of TRIS (Bq), a_TOT_ is the total used radioactivity (Bq), *t* is the incubation time (d), and the value 1.06 is the correction factor for the isotopic fractionation of sulfur (Jørgensen 1978). Since the samples were incubated with media in a slurry, we consider the results as “potential” SRR. The minimum quantification limit (MQL) and minimum detection limit (MDL) were calculated as follows:


\begin{eqnarray*}
\begin{array}{@{}*{1}{l}@{}} {{\mathrm{MDL}} = {\textrm{Average value of blank }}{{\mathrm{a}}_{{\mathrm{TRIS}}}}\left( {{\mathrm{KCs}},{\mathrm{MCs}},{\mathrm{DBc}},{\textrm{and CBs}}} \right)}\\ {{\mathrm{MQL}} = {\mathrm{MDL}} + {\mathrm{k}} \times \left( {{\textrm{standard deviation of blank }}{{\mathrm{a}}_{{\mathrm{TRIS}}}}} \right)\!,} \end{array}
\end{eqnarray*}

where k is a factor for a confidence level (Kaiser 1970). k = 3 was applied for the MQL and its confidence level is 95% instead of 99.86% as the blanks are non-normally distributed (Kaiser 1970). The a_TRIS_ of samples and blanks are compared to determine MDL and MQL.

## Results

### Hydrocarbon compositions in subsurface sediment

**Figure 2.**
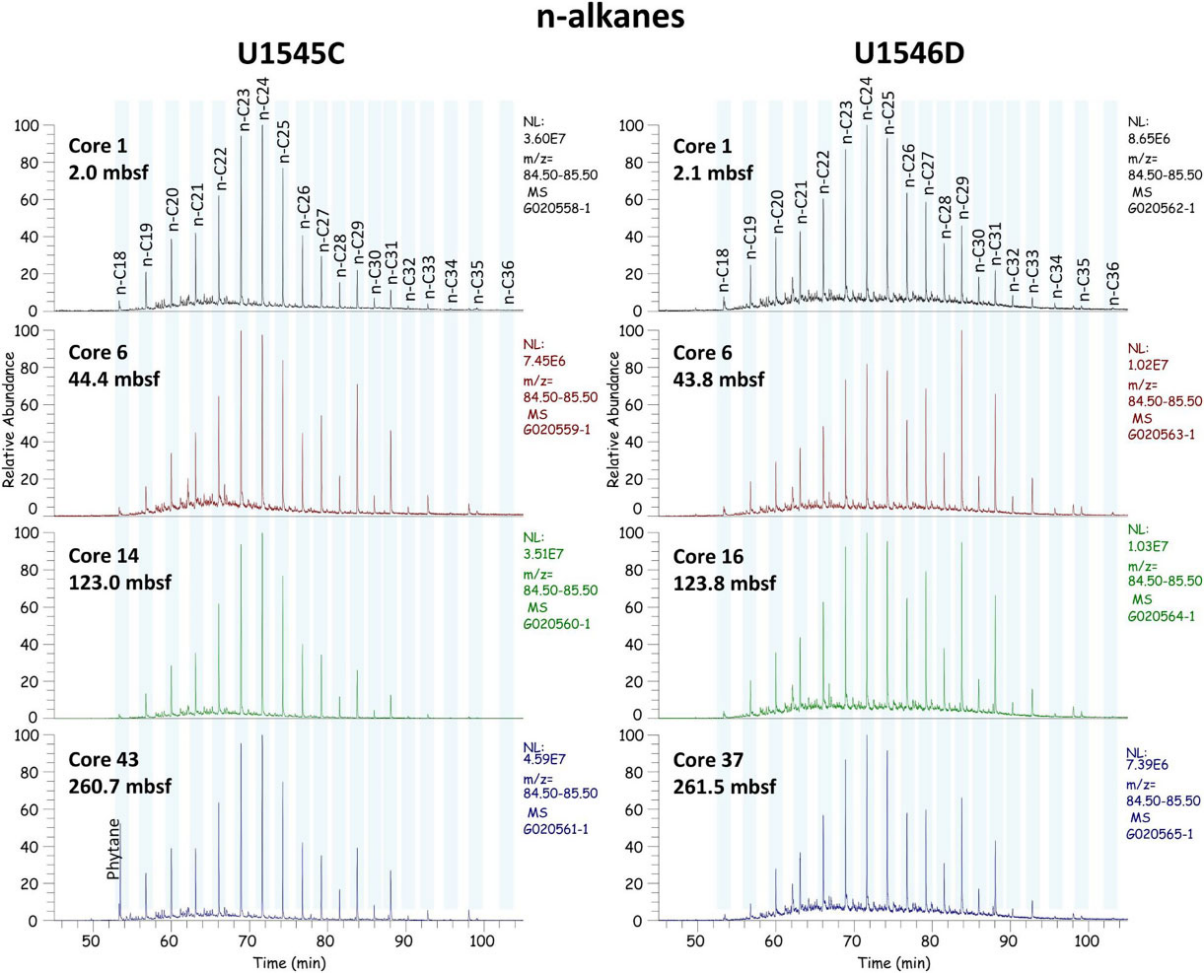
The *n*-alkane distribution of sediment samples from four different depth from the nonsill site (Site U1545C) and the sill site (U1546D) analyzed with GC–MS. The highest peak of each panel is set to 100% of the relative abundance.

GC–MS analyses revealed that the abundance of *n*-alkanes differs slightly with depth, but there is no clear difference between the two sites (Fig. 2). A homologue series of *n*-alkanes ranging from *n*-C_18_ to *n*-C_36_ was observed in all samples investigated, whereas *n*-alkanes longer than *n*-C_31_ hardly occur. The chromatograms show a bimodal alkane distribution with a dominant maximum around *n*-C_23_ or *n*-C_24_ with no odd over even carbon number predominance and a smaller maximum at *n*-C_29_ in the long chain range with a distinct odd over even carbon number predominance. Immature organic biomass contains *n*-alkanes with a strong odd over even carbon number predominance, which gets lost during geothermal maturation (Bray and Evans 1961). Thus, the *n*-alkane distribution in the Guaymas Basin sediments indicates that geothermally generated mature hydrocarbons (fossil hydrocarbons; no carbon number predominance) overprint the natural immature *n*-alkane signal (odd carbon number predominance), which is still visible in the long chain range around the maximum at *n*-C_29_ (Mangelsdorf and Rullkötter 2003). The immature *n*-alkane signal, especially visible in Core 6 samples (Fig. 2), represents the typical *n*-alkane distribution from higher land plants from the adjacent continent (Eglinton and Hamilton 1967). Fossil hydrocarbons dominate in particular in the surface near Core 1 at both sites.

Additionally, to the aliphatic hydrocarbon fraction the polar compound fraction containing NSO-containing compounds (NSO fraction) were analyzed using FT–ICR–MS. This analysis was done, because the free hetero-atomic biomass represents a good substrate source for microbial degradation, due to the energetically higher vulnerability of the functional group in the hydrocarbon structure. The FT–ICR–MS technique allows to characterize the polar compounds by their elemental composition and to order them into specific compound groups according to their hetero-atoms and thus, providing an insight into the compositional diversity of the biomass.

**Figure 3.**
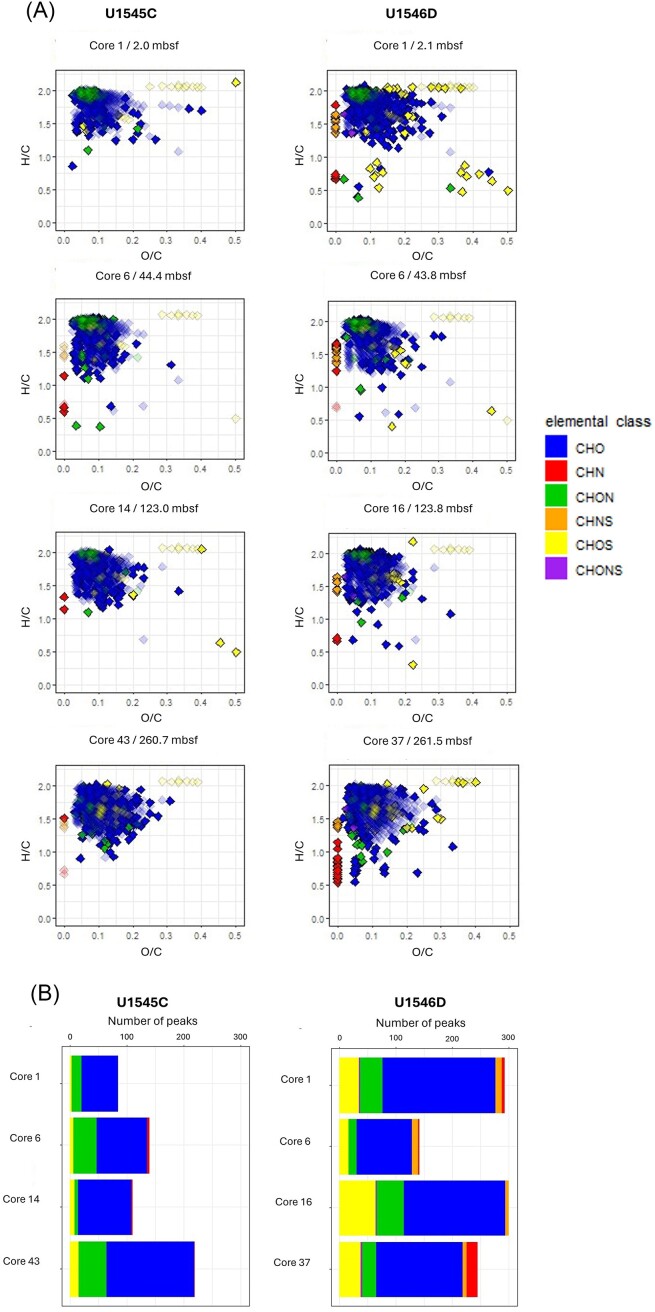
(A) Van Krevelen diagram of organic compounds extracted from four different depths from the nonsill site (U1545C) and the sill site (U1546D). The color codes represent the different heteroatomic groups within the organic compounds. Unique compounds of each sample are indicated with nontransparent symbols while shared signals are transparent. (B) Number and elemental class affiliation of unique compounds of each sample.

The compositional data are presented in Van Krevelen diagrams, in which each molecular formula detected is plotted according to its O/C and H/C ratios (Fig. 3A). The data have been processed to a Venn analysis before identifying unique and common compounds in the same depths of the nonsill site and the sill site. Common compounds of each depth are plotted as transparent symbols, while unique compounds are shown in solid colors. The data show mainly six different elemental classes indicated by different colors: CHO, CHN, CHON, CHNS, CHOS, and CHONS compounds. The main elemental class is the CHO compound group, with carboxylic acids representing the dominant proportion (60%–72% of CHO). Comparing both sites, the sill site showed a higher compositional diversity of unique compounds and with exception of Core 6 of the sill site also a greater number of unique compounds than the same depth of the nonsill site (Fig. 3B). The CHN compounds (red data points) in the deepest sample of the sill site represent carbazoles. Their presence as well as other compounds with low H/C values, inferring higher aromaticity, indicate fossil carbon compounds, presumably migrated from greater depth.

### Detection of hydrocarbon and inorganic nitrogen uptake with NanoSIMS

**Figure 4.**
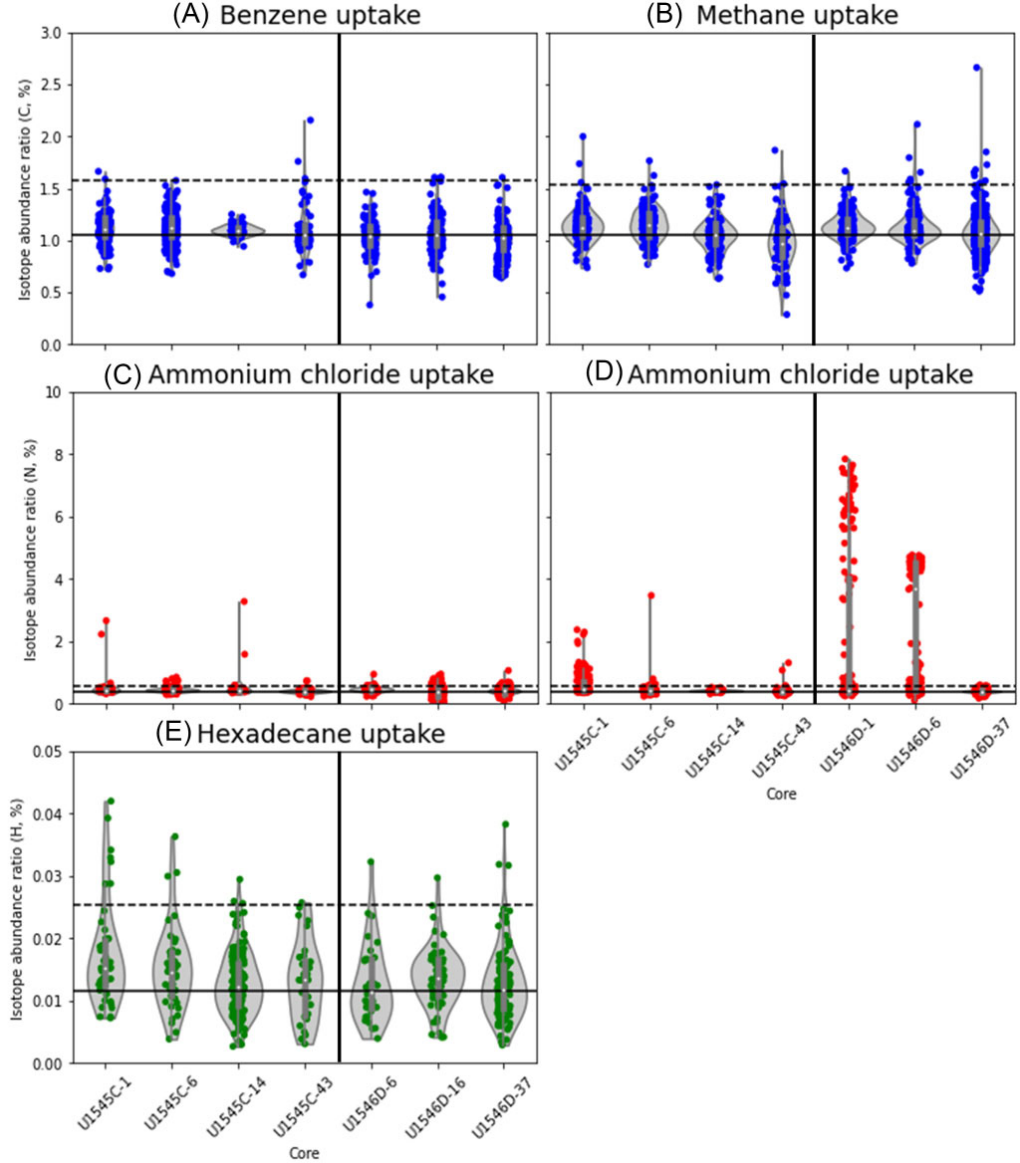
Isotope abundance ratios of each sample. (A and B) Carbon uptake from benzene (A) and methane (B). (C and D) Nitrogen uptake from ammonium chloride. (E) Hydrogen uptake from *n*-hexadecane. (A, C, and E) Samples incubated with benzene and *n*-hexadecane. (B and D) Samples incubated with methane. KCs were incubated with ^15^N-ammonium chloride plus either with benzene and *n*-hexadecane (A, C, and E) or with methane (B and D). Black solid lines represent the natural incorporation ratios of each isotope (carbon = 1.06%, nitrogen = 0.4%, and hydrogen = 0.0115%, respectively). Black dashed lines represent the statistical threshold values of the uptake of respective isotopes (A, C, and E: carbon = 1.58%, nitrogen = 0.58%, and hydrogen = 0.0255%, respectively; B and D: carbon = 1.54%, and nitrogen = 0.57%, respectively). See the method section for more details. A few data are missing due to the breaking of vials during incubation and the lack of additional sediment to repeat the experiments. Note the different ranges of isotope abundance ratios of each element on the *y*-axes. (Raw value data are described in Supplementary data S1–S4.)

We compared hydrocarbon uptake in the incubated samples with those from the KCs to determine if the uptake was statistically significant (Fig. 4). The vast majority of the ROI indicates no uptake of carbon compounds (Fig. 4A and B). However, a few ROI indicate low levels of benzene uptake (Fig. 4A) and visible uptake of carbon from methane (Fig. 4B).

Nitrogen from ammonium chloride was assimilated in almost all samples, especially in Core 1 and Core 6 from the sill site in incubations with methane (Fig. 4C and D). The highest isotope abundance ratio of Core 1 from the sill site incubated with methane reaches more than 8%. The median isotope abundance ratio of ROI of Core 6 from the sill site incubated with methane shows about 4%.

Since we added benzene and *n*-hexadecane simultaneously in the incubations, the uptake of *n*-hexadecane was observed by deuterium incorporation (Fig. 4E). The isotope abundance ratio of the nonsill site apparently decreases with depth, and hence with increasing temperature, though only comparatively few ROI are above the statistically significant value.

As uptake of carbon from benzene or methane and nitrogen from ammonium chloride was analyzed simultaneously with NanoSIMS, we checked if they were taken up simultaneously. At the nonsill site, only one cell out of 177 cells metabolized both methane and ammonium chloride in Core 6, and another cell out of 56 cells metabolized both benzene and ammonium chloride in Core 43. At the sill site, two cells out of 141 cells metabolized both methane and ammonium chloride in Core 6, and one cell out of 112 cells metabolized both benzene and ammonium chloride in Core 16. This data means that the vast majority of cells did not metabolize two labeled compounds simultaneously.

Overall, the results show a small but detectable uptake of hydrocarbons (benzene, *n*-hexadecane, and methane) and inorganic nitrogen (ammonium chloride).

**Figure 5.**
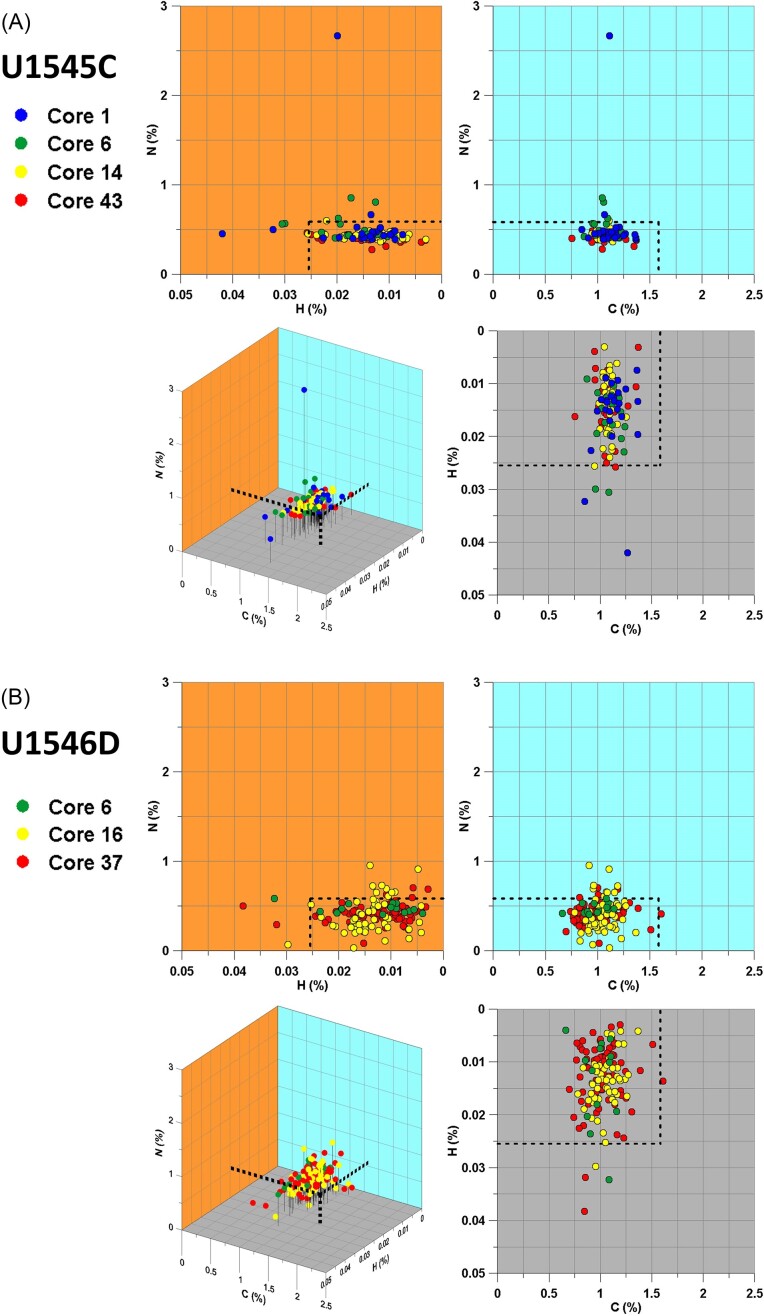
3D plots of isotope abundance ratios for carbon (benzene), nitrogen (ammonium chloride), and hydrogen (*n*-hexadecane) uptake. (A) the nonsill site (Site U1545C) and (B) the sill site (Site U1546D). Black dashed lines represent the thresholds for each element (C = 1.58%, N = 0.584%, and H = 0.0255%), above which incorporation becomes statistically significant corresponding to Fig. 4.

Based on the ^13^C/(^12^C+^13^C), ^12^C^15^N/(^12^C^14^N+^12^C^15^N), and ^2^H/(^1^H+^2^H) values from the identical ROI, we summarized the data in a 3D plot (Fig. 5). The figure shows only the results from the samples incubated with benzene and *n*-hexadecane as the samples incubated with methane were not incubated with a ^2^H-labeled substance.

Our results indicate that there is no simultaneous uptake of multiple isotope-labeled compounds, i.e. hydrogen from *n*-hexadecane, carbon from benzene, and nitrogen from ammonium chloride. The samples from near the seafloor tend to have higher incorporation ratios than those from deeper cores (Figs 4 and 5). There are two remarkable signals from the nonsill site Core 1 (Fig. 5A). One shows a high nitrogen isotope abundance ratio (≈ 2.67%) and another shows a high hydrogen isotope abundance ratio (≈ 0.0420%).

### SRR and the addition of hydrocarbons

**Figure 6.**
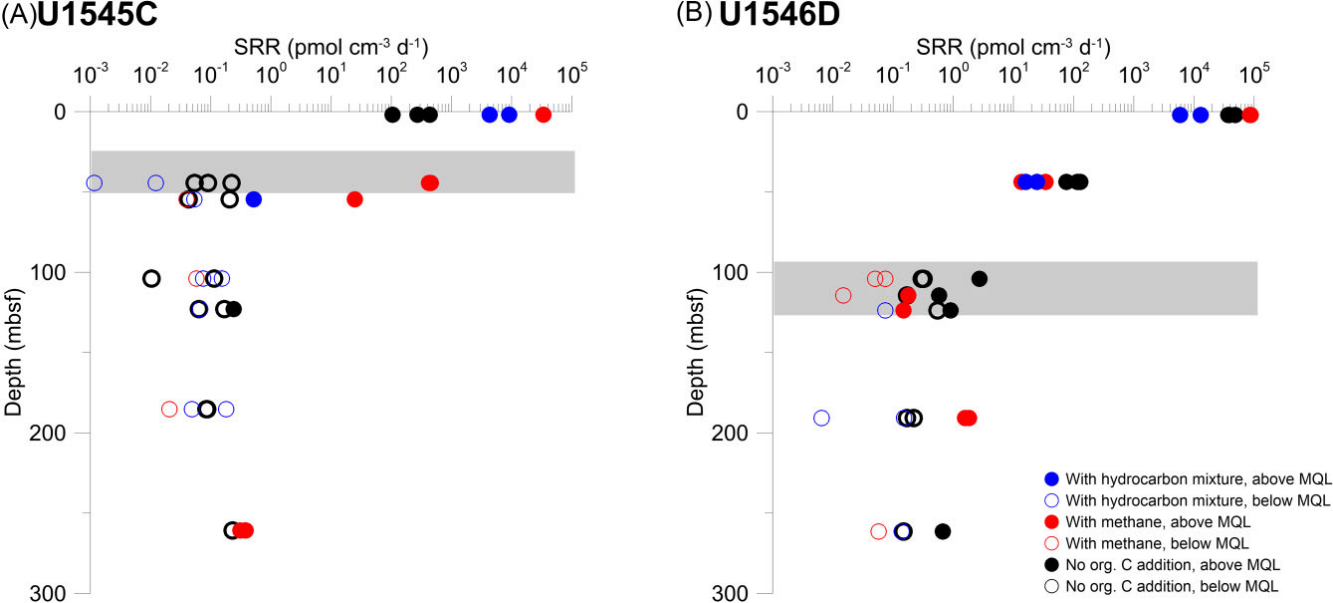
(A) Sulfate reduction rates (SRR) at the nonsill site (U1545C). (B) SRR at the sill site (U1546D). Blue circles show SRR measured from the samples incubated with the hydrocarbon mixture, and red circles show SRR measured from the samples incubated with methane. Black circles show SRR measured from the samples incubated without any substrate addition (Nagakura et al. 2022). Solid circles indicate SRR > MQL and open circles indicate SRR < MQL. Gray bars indicate the depth of the respective SMTZ (ca. 40 mbsf and ca. 110 mbsf, respectively). A few data points are missing due to the loss of vials during incubation. Due to the lack of sediment, these experiments could not be repeated. All incubations were carried out at *in situ* temperature and pressure. (Raw value data are described in Supplementary data S5.)

To examine the effect of hydrocarbon additions on microbial catabolic activity, we measured SRR in samples from both sites, the nonsill site (U1545C) and the sill site (U1546D), covering a wide depth and temperature range (from 2 mbsf to 261 mbsf; from 4°C to 63°C) (Fig. 6). The study of Nagakura et al. (2022) presented SRR measured on the same samples but without hydrocarbon additions, briefly, SRR were quantifiable whilst no organic substrate was amended and decreased with depth. At the nonsill site, SRR increased upon the addition of methane and the hydrocarbon mixture in the shallowest sample (2 mbsf, Core 1) (Fig. 6A). In Cores 6 and 7 near the SMTZ (44 mbsf and 55 mbsf, respectively), SRR increased upon the addition of methane, representing the increase of methanotrophic sulfate reduction (AOM), but not when adding the hydrocarbon mixture. In samples from below the SMTZ SRR did not increase at the nonsill site adding either methane or hydrocarbons. At the sill site SRR did not increase upon hydrocarbon addition (Fig. 6B). However, below the SMTZ at 191 mbsf (Core 23), SRR above MQL were observed upon the addition of methane, whereas without any substrate additions SRR fell below MQL.

## Discussion

### Relation between the assimilation of organic/inorganic matter and dissimilatory sulfate reduction

Our results indicate a greater uptake of deuterated *n*-hexadecane than ^13^C-benzene (Fig. 4A and E). However, given the different detection of the NanoSIMS for each isotope, we cannot draw any conclusions whether *n*-hexadecane was actually taken up preferentially over benzene. Edgcomb et al. (2022) showed that linear and nonbranched aliphatic hydrocarbons like *n-*hexadecane can be degraded more easily by microorganisms than benzene, which is an aromatic hydrocarbon, and thus more stable and recalcitrant.

In samples from the sill site Cores 1 and 6, nitrogen (from ammonium chloride) and methane-derived carbon were assimilated (Fig. 4B and D), but SRR did not increase with the addition of methane (Fig. 6B). We interpret this observation as sulfate-reducing microorganisms being active but they did not use methane because of the abundance of more favorable organic compounds produced by diagenetic alteration or geothermal maturation, triggered by the sill intrusion in the past (Teske et al. 2014), even though the heating from the sill at the sill site has already ceased (Teske et al. 2021a, Nagakura et al. 2022). Other electron acceptors such as NO_3_^−^, Mn(IV), or Fe(III) could in principle contribute to the assimilation, but these electron acceptors are depleted in the sediment and hence not available for microbial metabolisms (LIMS Reports: https://web.iodp.tamu.edu/LORE/, last accessed data: 11 April 2024). The significant ammonium uptake suggests that nitrogen source is either limited in these deep subsurface sediments and/or inorganic nitrogen is taken up faster than hydrocarbons. This observation concurs with the study of Morono et al. (2011), in which atomic ratios of nitrogen incorporation from ^15^N-ammonium were higher than those of carbon incorporations (^13^C-glucose, ^13^C-acetate, ^13^C-pyruvate, ^13^C-bicarbonate, and ^13^C-methane), Trembath-Reichert et al. (2017), in which ^15^N-ammonium and ^15^N-methylamine were incorporated although ^13^C-methanol and ^13^C-methylamine uptake was not observed, and Morono et al. (2020), in which nitrogen incorporation was generally more extensive than carbon incorporation and could already be observed within the first 21 days of incubation.

In Core 6 sample from the sill site, nitrogen uptake was higher when the sediment was incubated with methane (Fig. 4D) as compared to an incubation with a mixture of hydrocarbons (benzene and *n*-hexadecane) (Fig. 4C). This result might be due to either methane stimulating or benzene/*n*-hexadecane suppressing nitrogen uptake. Although the details remain elusive, nitrogen uptake by microorganisms in deep subsurface sediments is well-documented (Morono et al. 2011, 2020, Trembath-Reichert et al. 2017). According to the works by Kellermann et al. (2012), inorganic carbon (bicarbonate) is taken up preferentially over methane, but methane is used as an energy source, i.e. for catabolic metabolism.

Because microbial sulfate reduction is a catabolic process and more exergonic than anabolic hydrocarbon uptake, it can be measured in a shorter incubation experiment. Trembath-Reichert et al. (2017), for example, carried out anabolic process studies using anoxic sediments from off Shimokita peninsula, Japan, and incubated those samples for 2.5 years to reach a detectable signal of incorporation, while Glombitza et al. (2016) incubated sediment from the same drill core for only 10 days to achieve detectable SRR. We also found that 10 days was generally sufficient for SRR detection. Potential SRR in Glombitza et al. (2016) showed lower SRR than ours. This is probably also because they incubated their samples not at *in situ* pressure but just slightly above ambient conditions (∼2 bar). Since pressure can be an important factor to enhance microbial metabolic activity, we cannot simply compare the SRR data. But in general, our SRR data showed higher values. Although our samples for incorporation studies were incubated for only 42 days, which was relatively short, even the uptake of hydrocarbons was successfully observed. However, to observe a higher degree of assimilation, a longer incubation time (more than 1 year, for instance) would be needed.

Gene expression data from neighboring holes U1545B (nonsill site) and U1546B (sill site) showed differences between sites (Mara et al. 2023). Gene expressions related to several energy-related metabolisms and methanogenesis at Site U1546B are higher than at Site U1545B. In general, Site U1545B showed little gene expression associated with methane cycling and chemoautotrophy. Although their data were obtained from the original core samples, which are different from our data after incubation of 10 days (for SRR measurements) or 42 days (for NanoSIMS substrate uptake analyses), the different gene expression levels provide an explanation for the different responses of metabolic activities in the nonsill site and the sill site. Nonetheless, gene expression levels related to sulfate reduction are very low at both sites (Mara and Edgcomb, personal communication).

### Anaerobic degradation of hydrocarbons and sulfate-reducing microorganisms

It has been already known for decades that sulfate-reducing microorganisms can degrade both aliphatic and aromatic hydrocarbons under anaerobic conditions (e.g. Rueter et al. 1994, Meckenstock and Mouttaki 2011, and references therein) despite their molecular stability. Given the vast literature on this topic, it is safe to assume that sulfate-reducing microorganisms in the Guaymas Basin sediment can degrade the aromatic hydrocarbons used in this study: benzene (Edwards and Grbić-Galić 1992, Lovley et al. 1995, Anderson and Lovley 2000), naphthalene (Zhang and Young 1999, Meckenstock et al. 2000, Musat et al. 2009), phenanthrene (Zhang and Young 1999, Ramsay et al. 2003, Davidova et al. 2007, Tsai et al. 2009), and anthracene (Ramsay et al. 2003). Furthermore, the GC–MS data confirm that fossil hydrocarbons are an integral part of the biomolecule diversity in the sediments of Guaymas Basin (Fig. 2) most likely introduced by seeps on the seafloor or eroded source rocks during time of sediment deposition. Therefore, we assume that the increase in SRR was induced by one or several of the added aliphatic/aromatic hydrocarbons. With the exception of methane, all eight hydrocarbons (four aliphatic and four aromatic hydrocarbons) were added simultaneously, so it is not possible to identify which one was used for dissimilatory sulfate reduction.

The samples from the sill site showed a less positive SRR response to the hydrocarbon addition compared to the samples from the nonsill site (Fig. 6). This result may be because the sulfate-reducing microorganisms at the sill site did not metabolize as much of the added hydrocarbons as at the nonsill site, although cell abundances between these two sites are similar (∼10^9^ cells cm^−3^ near the seafloor to ca. 10^6^ cells cm^−3^ around 175 mbsf) (Teske et al. 2021b, c). One possible explanation for these findings might be related to the sill emplaced at the sill site and its impact on the deposited organic matter. Upon heating, macromolecular sedimentary organic carbon might have been pyrolyzed and converted into bioavailable organic substrates in the past (Horsfield et al. 2006), so the input of heat into the sedimentary system of the sill site (Teske et al. 2014), might have increased the diversity and availability of microbial substrates. Given the overall low microbial activity in these sediments, especially at greater depths, and the heat sterilization of the sediment around the intruded sill as well as the subsequent recolonization of the sediment, the microbial substrates that were produced by thermal cracking might be still available in the sediments, even though the heat effect of the sill at the sill site has already ceased. The extent of the thermal aureole and hence the sterilized zone is still unknown, but the TOC data show that a decrease in TOC concentration due to conversion into volatile or at least mobile compounds is restricted to about 24–37 m above and below the sill, respectively (Lizarralde et al. 2023). Over the depth range covered by our samples (ca. 2–260 mbsf at both sites) TOC concentrations scatter ~1%–2.5% with no visible trends. As our deepest sample was from about 100 m above the sill, it was well above the estimated zones of oil or gas generation (Lizarralde et al. 2023), so we assume no direct influence by the heat from the sill. However, Lizarralde et al. (2023) estimated that 3.3 Mt of sedimentary carbon was released by this sill intrusion via thermal cracking of the kerogen. The released carbon would be qualitatively different and potentially more bioavailable (Horsfield et al. 2006). If those thermogenically produced bioavailable carbon sources are still present in the sediment, even at very low concentrations, they would provide a reservoir of readily bioavailable substrates for the small microbial community (Mara et al. 2023), and thus the more recalcitrant hydrocarbons added in our experiment were not utilized, and hence did not lead to an increase in SRR. The data obtained from the FT–ICR–MS analyses reveal carbazoles (heterocyclic aromatic compounds consisting of two benzo groups attached to a central pyrrole ring) in the deepest sample of the sill site indicating that this site might have been influenced by migration of hydrocarbons from below. Furthermore, the sill site reveals a more diverse composition of organic compounds with different hetero-atoms or hetero-atom combinations (Fig. 3). Thus, the FT–ICR–MS data organic matter in these subsurface sediments differs qualitatively between sites, as they reveal different bioavailability of organic compounds with variable functional groups. These results might, therefore present a possible explanation for the differences in uptake of isotopically labeled compounds. The greater diversity and abundance of potentially bioavailable organic compounds at the sill site, therefore provide more favorable substrates than the added isotopically labeled compounds.

Comparing our organic geochemistry data (Figs 2 and 3), the anabolic data from our NanoSIMS analyses (Figs 4 and 5), and SRR data (Fig. 6) as well as the previously published molecular biological data (Mara et al. 2023), we argue that microorganisms can utilize hydrocarbons catabolically and anabolically but use them preferentially for catabolism.

## Summary

Microbial anabolic and catabolic utilization of various hydrocarbons in hydrothermally influenced subsurface sediments of Guaymas Basin were analyzed using incubation with stable- and radioisotope-labeled compounds as well as organic geochemical analyses. NanoSIMS analysis revealed the uptake of carbon from benzene and methane and hydrogen from *n*-hexadecane by microorganisms in a relatively short incubation time of 42 days. Nitrogen uptake from ammonium chloride was recorded especially from Cores 1 and 6 at the sill site, incubated together with methane, indicating the presence of active hydrocarbon-metabolizing processes. Radioisotope experiments showed an increase in SRR in samples from near the seafloor at the nonsill site (Site U1545C) upon the addition of hydrocarbons, whereas SRR did not increase in most samples from the sill site (Site U1546D). This result may infer that at the sill site more favorable carbon compounds are available, probably linked to the heating effect of a deeper-located sill intrusion, which had been active in the past. Overall, despite the low rate of turnover and substrate incorporation in deep subseafloor sediments of Guaymas Basin, our study shows that the indigenous microorganisms can utilize hydrocarbons for both anabolism and catabolism even in the deep subsurface of Guaymas Basin.

## Supplementary Material

fiae093_Supplemental_Files

## References

[bib1] Anderson RT, Lovley DR. Anaerobic bioremediation of benzene under sulfate-reducing conditions in a petroleum-contaminated aquifer. Environ Sci Technol. 2000;34:2261–6.

[bib2] Boetius A, Ravenschlag K, Schubert CJ et al. A marine microbial consortium apparently mediating anaerobic oxidation of methane. Nature. 2000;407:623–6. 11034209 10.1038/35036572

[bib3] Bray E, Evans E. Distribution of *n*-paraffins as a clue to recognition of source beds. Geochim Cosmochim Acta. 1961;22:2–15.

[bib4] Calvert S . Accumulation of diatomaceous silica in the sediments of the Gulf of California. Geol Soc Am Bull. 1966;77:569–96.

[bib5] Coates JD, Anderson RT, Lovley DR. Oxidation of polycyclic aromatic hydrocarbons under sulfate-reducing conditions. Appl Environ Microbiol. 1996;62:1099–101. 16535261 10.1128/aem.62.3.1099-1101.1996 PMC1388818

[bib6] Colwell FS, D'Hondt S. Nature and extent of the deep biosphere. Rev Mineral Geochem. 2013;75:547–74.

[bib7] Curray J, Moore D, Aguayo E et al. Leg 64: seeks evidence on development of basins. Geotimes. 1979;24:18–20.

[bib8] Davidova IA, Gieg LM, Duncan KE et al. Anaerobic phenanthrene mineralization by a carboxylating sulfate-reducing bacterial enrichment. ISME J. 2007;1:436–42. 18043662 10.1038/ismej.2007.48

[bib9] D'Hondt S, Jørgensen BB, Miller DJ et al. Distributions of microbial activities in deep subseafloor sediments. Science. 2004;306:2216–21. 15618510 10.1126/science.1101155

[bib10] Edgcomb VP, Teske AP, Mara P. Microbial hydrocarbon degradation in Guaymas Basin-exploring the roles and potential interactions of fungi and sulfate-reducing bacteria. Front Microbiol. 2022;13:831828–. 35356530 10.3389/fmicb.2022.831828 PMC8959706

[bib11] Edwards EA, Grbić-Galić D. Complete mineralization of benzene by aquifer microorganisms under strictly anaerobic conditions. Appl Environ Microbiol. 1992;58:2663–6. 1514813 10.1128/aem.58.8.2663-2666.1992 PMC195836

[bib12] Eglinton G, Hamilton RJ. Leaf epicuticular waxes: the waxy outer surfaces of most plants display a wide diversity of fine structure and chemical constituents. Science. 1967;156:1322–35. 4975474 10.1126/science.156.3780.1322

[bib13] Glombitza C, Adhikari RR, Riedinger N et al. Microbial sulfate reduction potential in coal-bearing sediments down to ∼2.5 km below the seafloor off Shimokita Peninsula, Japan. Front Microbiol. 2016;7:1576. 27761134 10.3389/fmicb.2016.01576 PMC5051215

[bib14] Hoehler TM, Alperin MJ, Albert DB et al. Field and laboratory studies of methane oxidation in an anoxic marine sediment: evidence for a methanogen-sulfate reducer consortium. Global Biogeochem Cyc. 1994;8:451–63.

[bib15] Holmkvist L, Ferdelman TG, Jørgensen BB. A cryptic sulfur cycle driven by iron in the methane zone of marine sediment (Aarhus Bay, Denmark). Geochim Cosmochim Acta. 2011;75:3581–99.

[bib16] Horsfield B, Schenk HJ, Zink K et al. Living microbial ecosystems within the active zone of catagenesis: implications for feeding the deep biosphere. Earth Planet Sci Lett. 2006;246:55–69.

[bib17] Inagaki F, Hinrichs K-U, Kubo Y et al. Exploring deep microbial life in coal-bearing sediment down to ∼2.5 km below the ocean floor. Science. 2015;349:420–4. 26206933 10.1126/science.aaa6882

[bib18] Ito M, Messenger S. Isotopic imaging of refractory inclusions in meteorites with the NanoSIMS 50 L. Appl Surf Sci. 2008;255:1446–50.

[bib19] Iversen N, Jorgensen BB. Anaerobic methane oxidation rates at the sulfate-methane transition in marine sediments from Kattegat and Skagerrak (Denmark). Limnol Oceanogr. 1985;30:944–55.

[bib20] Jørgensen BB . A comparison of methods for the quantification of bacterial sulfate reduction in coastal marine sediments. Geomicrobiol J. 1978;1:11–27.

[bib21] Jørgensen BB . Mineralization of organic matter in the sea bed—the role of sulphate reduction. Nature. 1982;296:643–5.

[bib22] Jørgensen BB . Bacteria and marine biogeochemistry. In: Marine Geochemistry. Vol. 173. Berlin: Springer, 2000, 207.

[bib23] Kaiser H . Report for analytical chemists. II. Quantitation in elemental analysis. Anal Chem. 1970;42:26A–59a.

[bib24] Kallmeyer J, Ferdelman TG, Jansen K-H et al. A high-pressure thermal gradient block for investigating microbial activity in multiple deep-sea samples. J Microbiol Methods. 2003;55:165–72. 14500008 10.1016/s0167-7012(03)00138-6

[bib25] Kallmeyer J, Ferdelman TG, Weber A et al. A cold chromium distillation procedure for radiolabeled sulfide applied to sulfate reduction measurements. Limnol Oceanogr Methods. 2004;2:171–80.

[bib26] Kallmeyer J, Pockalny R, Adhikari RR et al. Global distribution of microbial abundance and biomass in subseafloor sediment. Proc Natl Acad Sci. 2012;109:16213–6. 22927371 10.1073/pnas.1203849109 PMC3479597

[bib27] Kallmeyer J, Smith DC, Spivack AJ et al. New cell extraction procedure applied to deep subsurface sediments. Limnol Oceanogr Methods. 2008;6:236–45.

[bib28] Kallmeyer J . Contamination control for scientific drilling operations. Adv Appl Microbiol. 2017;98:61–91. 28189155 10.1016/bs.aambs.2016.09.003

[bib29] Kawka OE, Simoneit BRT. Survey of hydrothermally-generated petroleums from the Guaymas Basin spreading center. Org Geochem. 1987;11:311–28.

[bib30] Kawka OE, Simoneit BRT. Hydrothermal pyrolysis of organic matter in Guaymas Basin: I. Comparison of hydrocarbon distributions in subsurface sediments and seabed petroleums. Org Geochem. 1994;22:947–78.

[bib31] Kellermann MY, Wegener G, Elvert M et al. Autotrophy as a predominant mode of carbon fixation in anaerobic methane-oxidizing microbial communities. Proc Natl Acad Sci. 2012;109:19321–6. 23129626 10.1073/pnas.1208795109 PMC3511159

[bib32] Kubota K, Morono Y, Ito M et al. Gold-ISH: a nano-size gold particle-based phylogenetic identification compatible with NanoSIMS. Syst Appl Microbiol. 2014;37:261–6. 24702906 10.1016/j.syapm.2014.02.003

[bib33] Lechene C, Hillion F, McMahon G et al. High-resolution quantitative imaging of mammalian and bacterial cells using stable isotope mass spectrometry. J Biol. 2006;5:20. 17010211 10.1186/jbiol42 PMC1781526

[bib34] Lizarralde D, Teske A, Höfig TW et al. Carbon released by sill intrusion into young sediments measured through scientific drilling. Geology. 2023;51:329–33.

[bib35] Lovley DR, Coates JD, Woodward JC et al. Benzene oxidation coupled to sulfate reduction. Appl Environ Microbiol. 1995;61:953–8. 16534979 10.1128/aem.61.3.953-958.1995 PMC1388378

[bib36] Luque de Castro MD, Priego-Capote F. Soxhlet extraction: past and present panacea. J Chromatogr A. 2010;1217:2383–9. 19945707 10.1016/j.chroma.2009.11.027

[bib37] Mangelsdorf K, Rullkötter J. Natural supply of oil-derived hydrocarbons into marine sediments along the California continental margin during the late Quaternary. Org Geochem. 2003;34:1145–59.

[bib38] Mara P, Zhou Y-L, Teske A et al. Microbial gene expression in Guaymas Basin subsurface sediments responds to hydrothermal stress and energy limitation. ISME J. 2023;17:1907–19. 37658181 10.1038/s41396-023-01492-z PMC10579382

[bib39] Meckenstock RU, Annweiler E, Michaelis W et al. Anaerobic naphthalene degradation by a sulfate-reducing enrichment culture. Appl Environ Microbiol. 2000;66:2743–7. 10877763 10.1128/aem.66.7.2743-2747.2000 PMC92068

[bib40] Meckenstock RU, Mouttaki H. Anaerobic degradation of non-substituted aromatic hydrocarbons. Curr Opin Biotechnol. 2011;22:406–14. 21398107 10.1016/j.copbio.2011.02.009

[bib41] Morono Y, Inagaki F. Analysis of low-biomass microbial communities in the deep biosphere. Adv Appl Microbiol. 2016;95:149–78. 27261783 10.1016/bs.aambs.2016.04.001

[bib42] Morono Y, Ito M, Hoshino T et al. Aerobic microbial life persists in oxic marine sediment as old as 101.5 million years. Nat Commun. 2020;11:3626. 32724059 10.1038/s41467-020-17330-1 PMC7387439

[bib43] Morono Y, Terada T, Kallmeyer J et al. An improved cell separation technique for marine subsurface sediments: applications for high-throughput analysis using flow cytometry and cell sorting. Environ Microbiol. 2013;15:2841–9. 23731283 10.1111/1462-2920.12153 PMC3910163

[bib44] Morono Y, Terada T, Nishizawa M et al. Carbon and nitrogen assimilation in deep subseafloor microbial cells. Proc Natl Acad Sci. 2011;108:18295–300. 21987801 10.1073/pnas.1107763108 PMC3215001

[bib45] Morono Y . Accessing the energy-limited and sparsely populated deep biosphere: achievements and ongoing challenges of available technologies. Progr Earth Planet Sci. 2023;10:18.

[bib46] Musat F, Galushko A, Jacob J et al. Anaerobic degradation of naphthalene and 2-methylnaphthalene by strains of marine sulfate-reducing bacteria. Environ Microbiol. 2009;11:209–19. 18811643 10.1111/j.1462-2920.2008.01756.x

[bib47] Nagakura T, Schubert F, Wagner D et al. Biological Sulfate Reduction in Deep Subseafloor Sediment of Guaymas Basin. Front Microbiol. 2022;13:845250. 35308366 10.3389/fmicb.2022.845250 PMC8927301

[bib49] Parkes RJ, Cragg B, Roussel E et al. A review of prokaryotic populations and processes in sub-seafloor sediments, including biosphere:geosphere interactions. Mar Geol. 2014;352:409–25.

[bib50] Radke M, Willsch H, Welte DH. Preparative hydrocarbon group type determination by automated medium pressure liquid chromatography. Anal Chem. 1980;52:406–11.

[bib51] Ramsay JA, Li H, Brown RS et al. Naphthalene and anthracene mineralization linked to oxygen, nitrate, Fe(III) and sulphate reduction in a mixed microbial population. Biodegradation. 2003;14:321–9. 14571949 10.1023/a:1025620710581

[bib52] Rueter P, Rabus R, Wilkest H et al. Anaerobic oxidation of hydrocarbons in crude oil by new types of sulphate-reducing bacteria. Nature. 1994;372:455–8. 7984238 10.1038/372455a0

[bib53] Schippers A, Neretin LN, Kallmeyer J et al. Prokaryotic cells of the deep sub-seafloor biosphere identified as living bacteria. Nature. 2005;433:861–4. 15729341 10.1038/nature03302

[bib54] Shin B, Kim M, Zengler K et al. Anaerobic degradation of hexadecane and phenanthrene coupled to sulfate reduction by enriched consortia from northern Gulf of Mexico seafloor sediment. Sci Rep. 2019;9:1239. 30718896 10.1038/s41598-018-36567-x PMC6361983

[bib55] Teske A, Callaghan AV, LaRowe DE. Biosphere frontiers of subsurface life in the sedimented hydrothermal system of Guaymas Basin. Front Microbiol. 2014;5:362. 25132832 10.3389/fmicb.2014.00362 PMC4117188

[bib56] Teske A, Lizarralde D, Höfig T et al. Guaymas Basin tectonics and biosphere, International Ocean Discovery Program. Site U1545. College Station: IOPD Publications, 2021a.

[bib57] Teske A, Lizarralde D, Höfig T et al. Expedition 385 summary. In: Proceedings of the International Ocean Discovery Program. College Station: IOPD Publications, 2021b.

[bib58] Teske A, Lizarralde D, Höfig T et al. Guaymas Basin tectonics and biosphere, International Ocean Discovery Program. Site U1546. College Station: IOPD Publications, 2021c.

[bib59] Trembath-Reichert E, Morono Y, Ijiri A et al. Methyl-compound use and slow growth characterize microbial life in 2-km-deep subseafloor coal and shale beds. Proc Natl Acad Sci. 2017;114:E9206–15. 29078310 10.1073/pnas.1707525114 PMC5676895

[bib60] Trivedi MK, Branton A, Trivedi D et al. Determination of isotopic abundance of 13C/12C or 2H/1H and 18O/16O in biofield energy treated 1-chloro-3-nitrobenzene (3-CNB) using gas chromatography-mass spectrometry. Sci J Anal Chem. 2016;4:42–51.

[bib61] Tsai J-C, Kumar M, Lin J-G. Anaerobic biotransformation of fluorene and phenanthrene by sulfate-reducing bacteria and identification of biotransformation pathway. J Hazard Mater. 2009;164:847–55. 18848395 10.1016/j.jhazmat.2008.08.101

[bib62] Wagner M . Single-cell ecophysiology of microbes as revealed by Raman microspectroscopy or secondary ion mass spectrometry imaging. Annu Rev Microbiol. 2009;63:411–29. 19514853 10.1146/annurev.micro.091208.073233

[bib63] Wellsbury P, Goodman K, Barth T et al. Deep marine biosphere fuelled by increasing organic matter availability during burial and heating. Nature. 1997;388:573–6.

[bib64] Widdel F, Bak F. Gram-negative mesophilic sulfate-reducing bacteria. In: The Prokaryotes. Vol. 3352. Berlin: Springer, 1992, 3378.

[bib65] Zhang X, Young LY. Carboxylation as an initial reaction in the anaerobic metabolism of naphthalene and phenanthrene by sulfidogenic consortia. Appl Environ Microbiol. 1999;65:2279. 10.1128/aem.63.12.4759-4764.1997 PMC168798 9471963

